# Myosin Light Chain Kinase Mediates Intestinal Barrier Disruption following Burn Injury

**DOI:** 10.1371/journal.pone.0034946

**Published:** 2012-04-18

**Authors:** Chuanli Chen, Pei Wang, Qin Su, Shiliang Wang, Fengjun Wang

**Affiliations:** 1 State Key Laboratory of Trauma, Burns, and Combined Injury, Institute of Burn Research, Third Military Medical University, Southwest Hospital, Chongqing, China; 2 Department of Military Nursing, School of Nursing, Third Military Medical University, Chongqing, China; University of Chicago, United States of America

## Abstract

**Background:**

Severe burn injury results in the loss of intestinal barrier function, however, the underlying mechanism remains unclear. Myosin light chain (MLC) phosphorylation mediated by MLC kinase (MLCK) is critical to the pathophysiological regulation of intestinal barrier function. We hypothesized that the MLCK-dependent MLC phosphorylation mediates the regulation of intestinal barrier function following burn injury, and that MLCK inhibition attenuates the burn-induced intestinal barrier disfunction.

**Methodology/Principal Findings:**

Male balb/c mice were assigned randomly to either sham burn (control) or 30% total body surface area (TBSA) full thickness burn without or with intraperitoneal injection of ML-9 (2 mg/kg), an MLCK inhibitor. *In vivo* intestinal permeability to fluorescein isothiocyanate (FITC)-dextran was measured. Intestinal mucosa injury was assessed histologically. Tight junction proteins ZO-1, occludin and claudin-1 was analyzed by immunofluorescent assay. Expression of MLCK and phosphorylated MLC in ileal mucosa was assessed by Western blot. Intestinal permeability was increased significantly after burn injury, which was accompanied by mucosa injury, tight junction protein alterations, and increase of both MLCK and MLC phosphorylation. Treatment with ML-9 attenuated the burn-caused increase of intestinal permeability, mucosa injury, tight junction protein alterations, and decreased MLC phosphorylation, but not MLCK expression.

**Conclusions/Significance:**

The MLCK-dependent MLC phosphorylation mediates intestinal epithelial barrier dysfunction after severe burn injury. It is suggested that MLCK-dependent MLC phosphorylation may be a critical target for the therapeutic treatment of intestinal epithelial barrier disruption after severe burn injury.

## Introduction

It is well known that the intestinal epithelial mucosa plays a pivotal role in the host's protection against luminal pathogens and antigenic molecules, providing a barrier function to protect against the invasion of intraluminal microorganisms and endotoxin through the intestinal wall into the blood or lymph. However, it is also well documented that the intestinal epithelial barrier function is often disrupted in many surgical diseases, including trauma, shock, burn injury, and the other surgically critical illness, resulting in the increased intestinal permeability and subsequent translocation of bacteria or/and endotoxin from gastrointestinal tract [1], [2]. It has been recognized that increased gut permeability and bacteria or/and endotoxin translocation plays a very important role in the setting of severe complications such as systemic inflammatory response syndrome, sepsis, multiple organ dysfunction syndrome, and multiple organ failure. Therefore, the gastrointestinal tract has been believed to be a central organ or a motor of multiple organ dysfunctions after surgical stress [3], [4]. Thus, understanding the mechanisms of intestinal barrier disruption and maintaining the intestinal barrier function are critical to the clinically comprehensive treatment of severe burn victims.

An intact intestinal barrier is maintained through tight junctions, which are the key elements of the paracellular space, and intercellular junctions, which assist to seal the paracellular space between adjacent intestinal epithelial cells [5], [6]. The tight junction, composed of multiple proteins including transmembrane proteins such as zonula occludens (ZO), occludin, claudins and junctional adhesion molecule, is a complex that forms a selectively permeable seal between adjacent epithelial cells [5], [6]. Thus, tight junction opening is believed to be the key limiting factor of the intestinal mucosal paracellular pathway. Accumulating evidences have indicated that tight junction opening is triggered by the phosphorylation of myosin light chain (MLC), which predominantly depends on myosin light chain kinase (MLCK) activation [7], [8]. The activated MLCK catalyzes the MLC phosphorylation, which in turn results in the contraction of peri-junctional actinomyosin filaments and the tight junction opening. For example, studies using an inducible active MLCK have shown that MLC phosphorylation alone driven by active MLCK is sufficient to induce increased tight junction permeability and also causes the breakdown of the tight junction structural proteins ZO-1 and occludin [9]. Therefore, the MLCK-dependent MLC phosphorylation pathway appears to be critical to the pathophysiologically disrupted intestinal barrier both *in vitro* and *in vivo*.

To date, the mechanisms contributing to intestinal barrier breakdown after severe burn injury have not yet been elucidated. However, a recent study has shown that pentoxifylline, a nonspecific phosphodiesterase inhibitor known to increase intracellular levels of cyclic AMP, is able to limit burn-induced intestinal barrier breakdown and intestinal inflammation, and to attenuate MLCK activation [10], suggesting that MLCK activation might be involved in intestinal barrier breakdown after burn injury. We hypothesized that severe burn injury would activate the MLCK-dependent MLC phosphorylation pathway, which in turn lead to intestinal barrier breakdown, and that direct inhibition of MLCK activation would attenuate the burn-induced intestinal barrier disruption. Thus, in this study, we investigate the role of MLCK-dependent MLC phosphorylation signaling pathway in intestinal barrier breakdown induced by severe cutaneous thermal injury. Our present data demonstrate that MLCK-dependent MLC phosphorylation mediates intestinal epithelial barrier dysfunction after severe burn injury.

## Materials and Methods

### Ethics statement

The animal studies were approved by the Animal Care and Use Committee of the Third Military Medical University, and all the protocols were approved by the Ethics Committee of Southwest Hospital, Third Military Medical University, Chongqing, China.

### Animals

Healthy adult male balb/c mice weighing 20–25 g, supplied by the Animal Center, Third Military Medical University, were used in this study. The animals were housed in wire-bottomed, wire-lid cages, allowed access to chow and water ad libitum, and acclimatized for 1 week prior to experiments in a temperature-controlled room (25±2°C) with 12-hour light and dark cycles. Animals were randomly divided into 3 groups: control (sham burn), burn, and burn plus ML-9 treatment group.

### Burn model & experimental protocol

After acclimatization, mice assigned to burn group received a 30% total body surface area (TBSA) full thickness burn. Briefly, following a 12-h fast with water available ad libitum, animals were anesthetized with an intraperitoneal injection of sodium pentobarbital (30 mg/kg). Under anesthesia, the dorsal fur of the animals was shaved and depilated with 8% sodium sulfide. A 30% TBSA full-thickness flame burn was produced by igniting 3% napalm for 10 seconds on the depilated dorsum. Mice in control group received treatment similar to burn animals with the exception of the flame burn treatment, whereas the mice assigned to burn plus ML-9 treatment group were received an immediately intraperitoneal injection of ML-9 (2 mg/kg, Alexis Biochemicals, San Diego, CA) dissolved in saline after flame burn treatment. After injury, mice were immediately resuscitated with an intraperitoneal injection of 2.0 ml lactated Ringer's solution. Then, mice were individually caged, provided food and water *ad libitum*, and recovered from anesthesia. At the end of experiment, blood of the anesthetized mice from each experimental group was collected for intestinal permeability assay. Then, mice were sacrificed for tissue harvest. The harvested tissues were used for histological, immunofluorescent, and biochemical evaluation, as described blow.

### *In vivo* intestinal paracellular permeability assay

Intestinal paracellular permeability was determined by measuring the appearance in blood of a marker, 4.4 kDa fluorescein isothiocyanate-labeled dextran (FITC-dextran) (Sigma, St. Louis, MO). The assay of intestinal paracellular permeability was slightly modified from the previously described methods [11]–[13]. Briefly, a laparotomy was carried out under anesthesia before the animals were sacrificed at the end of experiment. A 5-cm segment of the ileum was dissociated beginning 5 cm proximal to the cecum, with well-protected superior mesenteric vessels. The bilateral end of the isolated ileum was ligated with 2-0 silk suture to prevent the leakage of FITC-dextran. 0.2 ml of 0.1 Mol/L phosphate buffer saline (PBS, pH 7.2) containing 20 mg of FITC-dextran was injected into the lumen, after which the laparotomy was closed. After 30 min, a blood sample was taken by cardiac puncture of the mice. The blood was centrifuged at 4°C, 3000 g for 10 min, and the plasma was taken for the analysis of FITC-dextran concentration. The plasma was diluted at 1∶10 with PBS, and then the fluorescence intensity of the diluted plasma was measured by using a fluorospectrophotometer (Hitachi Ltd, Tokyo, Japan) with an excitation wavelength of 480 nm and an emission wavelength of 520 nm. The plasma FITC-dextran concentrations were calculated from standard curves generated by serial dilution of FITC-dextran in PBS.

### Histological analysis

The ileal tissues were promptly rinsed with cold 0.9% saline solution and immediately fixed in 10% buffered formalin phosphate (pH 7.0) until processing for histological sections. The formalin-fixed samples were embedded in paraffin, and sectioned. After deparaffinization and dehydration, the sections were stained with hematoxylin and eosin for histological assessment of intestinal mucosa. Histological changes of intestinal mucosa were observed with a DM6000B microscope (Leica, Germany), and images were obtained using Metamorph 7.5 software (MDS Analytical Technologies, Downingtown, PA).

### Immunofluorescent staining, microscopy, and image analysis

Frozen sections of ileal tissue were fixed with 1% paraformaldehyde in PBS containing 1 mmol/L CaCl_2_ for 30 min at room temperature, then washed thrice with PBS for 5 min, permeabilized in 1% Triton X-100 in PBS at room temperature for 5 min. After washing with PBS, the nonspecific binding sites were blocked with 5% normal goat serum in PBS for 30 min. Then, the sections were incubated with monoclonal rabbit antibody against ZO-1, occludin or claudin-1 (Invitrogen, Carlsbad, CA) diluted at 1∶200 with 1% normal goat serum in PBS at 4°C overnight. After washing thrice in PBS, sections were incubated with secondary Alexa Fluor 488 conjugated goat anti-rabbit IgG antibody (Invitrogen) at 1∶100, Alexa Fluor 594-conjugated phalloidin (Invitrogen) at 5 U/ml, and DAPI (Sigma) for 1 hour at room temperature. After washing thrice in PBS, sections were mounted using Slowfade reagents (Invitrogen). Images were obtained using a TCS SP5 laser confocal microscopy (Leica, Germany).

### Immunoblot analyses of MLCK, MLC and phosphorylated MLC in intestinal mucosa

After the animals were sacrificed, a 5 cm ileal segment was taken to harvest mucosa by a glass slide. The harvested mucosa were homogenized with 10 volumes of ice-cold RIPA buffer containing 150 mM NaCl, 10 mM Tris-HCl, pH 7.5, 1% sodium deoxycholate, 1% NP-40, 10 mM EDTA, 0.1% SDS, including both protease and phosphatase inhibitor cocktail (Sigma), followed by a brief sonication with a sonicator (Tomy Seiko, Tokyo, Japan). Thereafter, the homogenates were centrifuged at 4°C, 15000 g for 10 min, and the supernatants were collected to determine protein concentration using the RC DC protein assay kit (Bio-Rad, Hercules, CA). For the determination of MLCK, MLC and pMLC, equal amounts of total protein extracted from the ileal mucosa were fractionated on 10% sodium dodecyl sulfate-polyacrylamide gel electrophoresis (SDS-PAGE) gel and then transferred to polyvinylidene difluoride (PVDF) membrane (Millipore, Bedford, MA). After complete transfer, membranes were blocked for 60 min at room temperature with 5% nonfat milk dissolved in TBST buffer. After blocking, membranes were incubated with antibodies specific for MLCK (1∶1000, Sigma), MLC (1∶2000, Sigma), phosphorylated MLC (pMLC, 1∶1000, Cell Signaling, Beverly, MA), and β-actin (1∶5000, Sigma) overnight at 4°C. After wash with TBST, membranes were incubated with appropriate peroxidase-conjugated secondary antibodies (Southern Biotech, Birmingham, AL) at room temperature for 60 min. The blots were visualized using Super Signal West Pico reagent (Pierce, Rockford, IL). The chemiluminescence signal was captured using a ChemiDoc XRS system (Bio-Rad). The densities of the bands were quantified with Quantity One Image software (Bio-Rad).

### Statistical analysis

Data are presented as means ± SEM. For multi-group data analysis, one-way analysis of variance (ANOVA) was performed using SPSS 13.0 statistical software. A *p* value of <0.05 was considered as the minimum level of significance in all cases. All reported significance levels represent two-tailed *p* values.

## Results

### MLCK inhibition with ML-9 decreases the burn-induced increase of intestinal permeability

**Figure 1:**
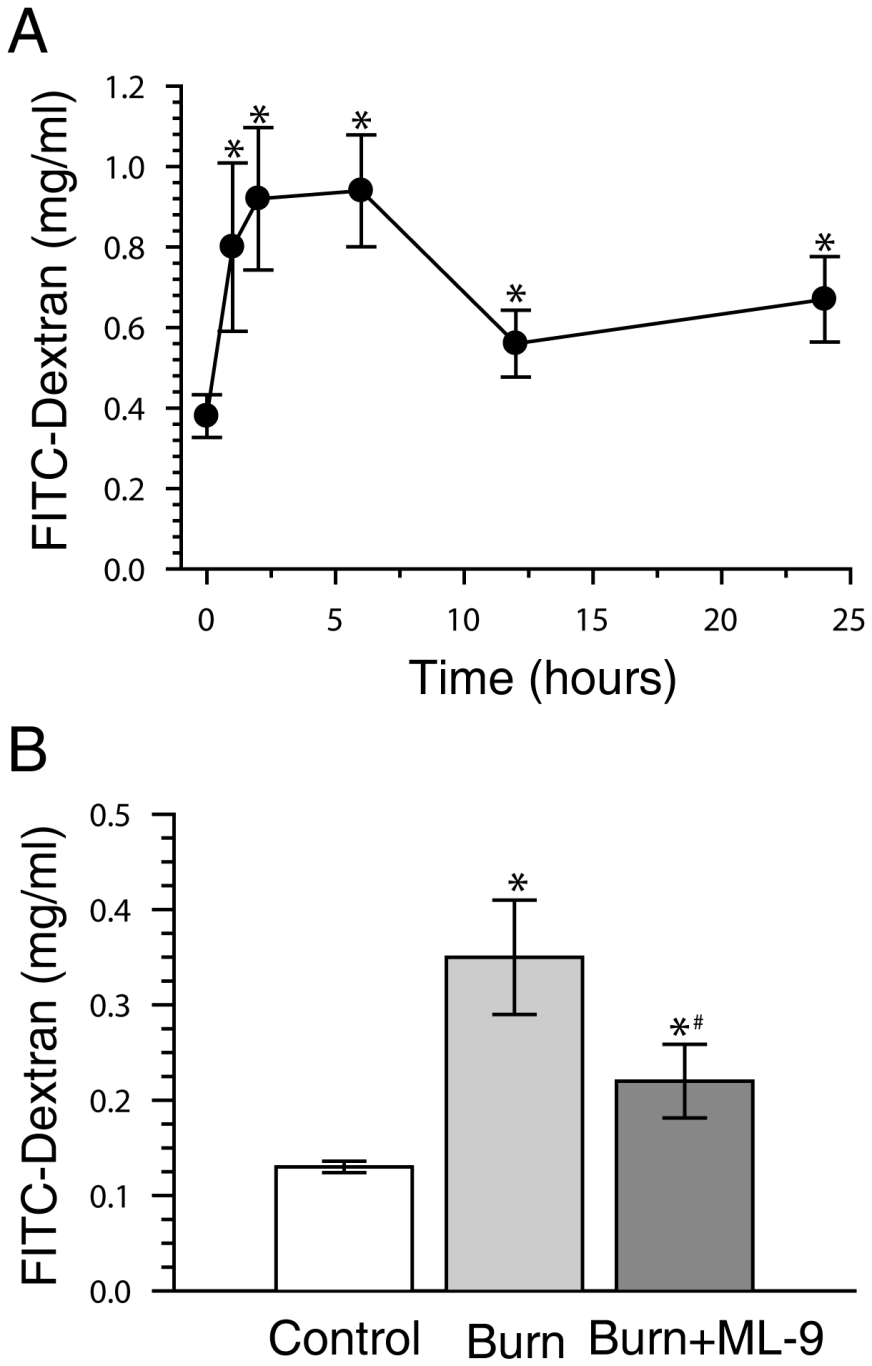
MLCK inhibition with ML-9 decreases the burn-induced increase of intestinal permeability. A. Intestinal permeability was assessed by measuring FITC-dextran in the systemic circulation after intraluminal injection of 4.4-kDa FITC-dextran at several time points after 30% TBSA burn injury. Intestinal permeability to 4.4-kDa FITC-dextran was significantly increased after burn injury. n = 7–10 per time point. *p<0.05 compared with 0 hour. B. Intestinal permeability was determined at 6 hours after 30% TBSA thermal injury. MLCK inhibition with ML-9 (2 mg/kg) decreases the burn-induced increase of intestinal permeability. n = 10 or 11 per group. *p<0.05 compared with control. #p<0.05 compared with burn.

By measuring the appearance in blood of 4.4 kDa FITC-dextran which was traditionally used to measure the movement of small molecules across the intestinal epithelium *in vivo*, we first did the time-course analysis of intestinal paracellular permeability in mice subjected to 30% TBSA full-thickness burn. As shown in Fig. 1A, when compared with that of control, the concentration of plasma FITC-dextran started to increase significantly at 1 hour following burn injury, peaked at 6 hour with approximately 3 folds of control, and still significantly higher than that of control at 24 h postburn. These results indicate that the intestinal paracellular permeability is significantly increased after severe cutaneous burn injury.

It has been well recognized that increased MLC phosphorylation triggered by activated MLCK is critical to intestinal barrier defect both *in vivo* and *in vitro* [7], [8]. Thus, based on the above data showing that the postburn intestinal permeability increased significantly and peaked at 6 h, we asked whether *in vivo* inhibiting MLC phosphorylation with ML-9, an MLCK inhibitor, could ameliorate the increased intestinal permeability induced by severe burn injury, and selected postburn 6 h as the time point for further studies. Our preliminary experiments proved that ML-9 alone had no obvious effect on intestinal permeability in normal balb/c mice (data not shown). As illustrated in Fig. 1B, although plasma FITC-dextran concentration in burn plus ML-9 treatment group was still a little bit higher than that in control group, it was significantly lower than that in burn group, indicating that *in vivo* inhibition of MLCK activity is capable of attenuating the increased intestinal permeability induced by severe burn injury.

### MLCK inhibition with ML-9 alleviates the histological damage of intestinal mucosa following burn injury

**Figure 2:**
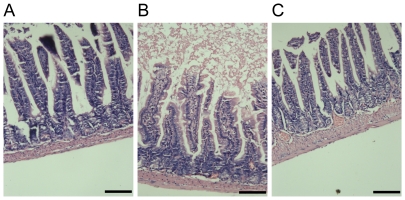
MLCK inhibition with ML-9 alleviates the histological damage of intestinal mucosa following burn injury. Hematoxylin and eosin staining of distal ileal segments was performed at 6 hours following 30% TBSA burn. A. Normal appearance was shown in the ileum from control mice. B. Histological damage characterized by loss of partial epithelial cells, exposed lamina propria, degeneration, necrosis, as well as signs of inflammatory cell infiltration was visualized in the ileum from burned mice. C. An appearance similar to control mice was shown in ileum from burned mice treated with ML-9, with near-normal appearing villi and a few of infiltrating inflammatory cells. Data are representative of five independent experiments. Scale bar = 100 µm.

As shown in Fig. 2A, histological examination of distal ileum showed normal appearing villi in mice of control group. However, the ileal mucosa tissue was seriously damaged in mice of burn group, which was characterized by loss of partial epithelial cells, exposed lamina propria, degeneration, necrosis, as well as signs of inflammatory cell infiltration at 6 h after burn injury (Fig. 2B). Sections from murine ileum of burn plus ML-9 treatment group showed an appearance similar to that of control group, with near-normal appearing villi, but still a few of infiltrating inflammatory cells (Fig. 2C). These data indicate that *in vivo* inhibition of MLCK activity with specific inhibitor attenuates the intestinal mucosa injury following severe burns.

### MLCK inhibition with ML-9 attenuates the burn-induced changes of tight junction proteins ZO-1, occludin and claudin-1

**Figure 3:**
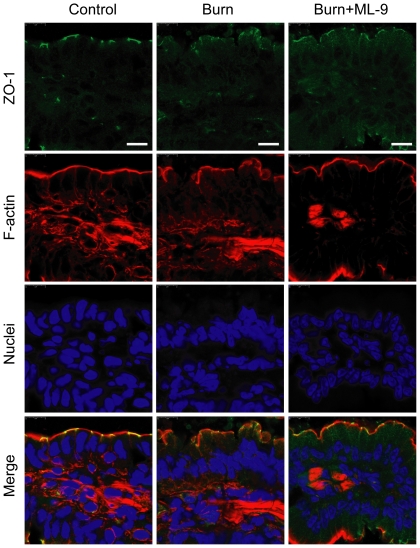
MLCK inhibition with ML-9 attenuates the burn-induced reorganization of tight junction protein ZO-1. Frozen sections of distal ileum were labeled for ZO-1 (green), F-actin (red) and nuclei (blue) at 6 hours after 30% TBSA burn. ZO-1 was localized to the epithelial tight junctions, and colocalized with the perijunctional F-actin ring in the ileum from control mice. ZO-1 was stained at the apical junctions in ileum from burned mice, with the loss of ZO-1 at the apical junctions. The burn-induced reorganization of ZO-1 was attenuated in the ileum from burned mice treated with ML-9. Data are representative of five independent experiments. Scale bar = 10 µm.

To more precisely understand the intestinal barrier defect induced by burn injury, we next evaluated the morphological changes of zonula occludens-1 (ZO-1), a member of tight junction proteins, by immunofluorescent antibody labeling assay. As shown in Fig. 3, in the ileum of mice from control group, ZO-1 was localized to the epithelial tight junctions, which is appreciated as a series of bright green spots at the apical compartment of cell-cell junctions. Meanwhile, ZO-1 was colocalized with the perijunctional filamentous (F)-actin ring labeled as red. In contrast, this ordered appearance of ZO-1 was disrupted in the ileum of mice from burn group, with the loss of bright green spots standing for ZO-1 at the apical junctions. However, the burn-induced redistribution of ZO-1 was attenuated by ML-9 treatment. These results indicate that the intestinal barrier function disruption is accompanied by the reorganization of tight junction protein ZO-1.

**Figure 4:**
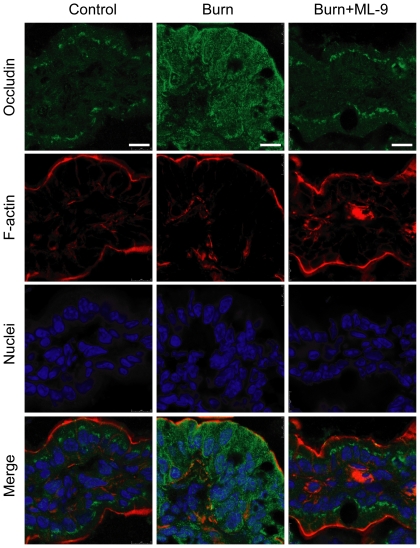
MLCK inhibition with ML-9 attenuates the burn-induced reorganization of tight junction protein occludin. Occludin was mainly localized to cytoplasm and epithelial tight junctions in the ileum from control mice. The burn-induced reorganization of occludin was attenuated in the ileum from burned mice treated with ML-9. Data are representative of five independent experiments. Scale bar = 10 µm.

**Figure 5:**
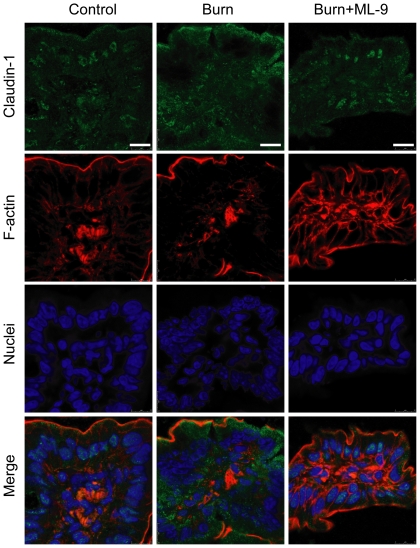
MLCK inhibition with ML-9 inhibits the burn-induced elevation of tight junction protein claudin-1. Claudin-1 was predominantly localized to cytoplasm in the ileum from control mice. Claudin-1 expression was markedly elevated in cytoplasm in the ileum from burned mice. The burn-induced increase of caludin-1 expression was inhibited by ML-9 treatment. Data are representative of five independent experiments. Scale bar = 10 µm.

Tight junction protein occludin was mainly localized in cytoplasm and epithelial tight junctions in the ileum of control mice (Fig. 4). After burn injury, occludin expression was markedly increased in cytoplasm and decreased in epithelial tight junctions. The changes of occludin expression was attenuated by ML-9 treatment. Unlike ZO-1 and occludin, claudin-1 was predominantly localized in cytoplasm in the ileum of control mice (Fig. 5). Caludin-1 expression was markedly elevated in cytoplasm following burn injury. The increase of cytoplasmic claudin-1 expression was inhibited by ML-9 treatment.

### MLCK inhibition with ML-9 abolishes the increase of MLC phosphorylation after burn injury

**Figure 6:**
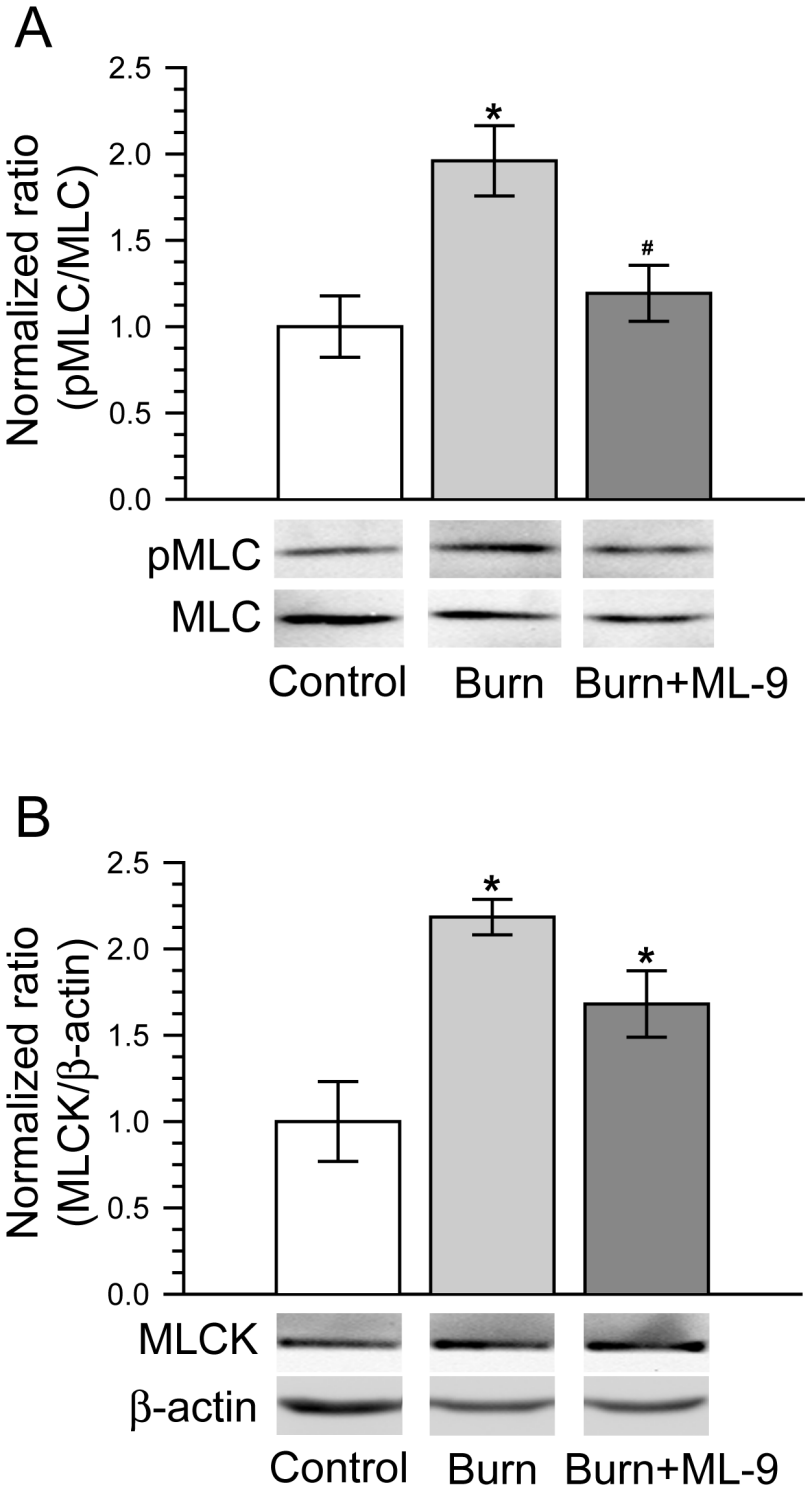
MLCK inhibition with ML-9 abolishes the increase of MLC phosphorylation after burn injury. A. Both phosphorylated MLC and total MLC in ileal mucosa were determined by Western blot at 6 hours after 30% TBSA burn. MLC phosphorylation was significantly increased following burn injury. MLCK inhibition with ML-9 abolishes the burn-caused increase of MLC phosphorylation. Data are representative of five similar experiments. *p<0.05 compared with control. #p<0.05 compared with burn. B. MLCK protein expression in ileal mucosa was analyzed by Western blot at 6 hours after 30% TBSA burn. Burn injury induced a significant increase of MLCK protein expression. ML-9 treatment had no significantly effect on the burn-induced increase of MLCK protein expression. Data are representative of five similar experiments. *p<0.05 compared with control.

It has been well documented that MLCK-dependent MLC phosphorylation is critical to intestinal barrier disruption [7], [8]. We and other investigators have previously demonstrated that increased MLC phosphorylation and MLCK protein expression are very important to intestinal epithelial barrier defect induced by proinflammatory cytokines [14]–[18]. We therefore considered the possibility that upregulation of MLCK-dependent MLC phosphorylation could be involved in the burn-induced defect of intestinal barrier function. MLC phosphorylation was assessed by immunoblot analyses of phosphorylated MLC. As shown in Fig. 6A, MLC phosphorylation of ileal mucosa increased significantly in burn group as compared with that in control group (p<0.05), whereas ML-9 treatment efficiently prevented the burn-induced increase of MLC phosphorylation.

MLCK has been proven to be the predominant determinant of MLC phosphorylation in intestinal epithelial cells [7], [8]. Thus, we next evaluated MLCK protein expression in ileal mucosa. As illustrated in Fig. 6B, a significant increase of MLCK expression was induced in burn group when compared with control group (p<0.05). However, ML-9 treatment had no significantly statistical effect on the burn-induced increase of MLCK protein expression in ileal mucosa.

## Discussion

In this study, it is demonstrated that severe burn injury causes the increase of intestinal permeability, and that the increased intestinal permeability induced by burn injury is accompanied by the histological damage of intestinal mucosa, redistribution of tight junction protein ZO-1 as well as upregulation of both MLCK protein and MLC phosphorylation. Our present study also reveals that MLCK inhibition with specific inhibitor ML-9 attenuates the burn-induced intestinal barrier disruption *in vivo*. These findings provide a new insight into the mechanisms involved in the intestinal barrier breakdown caused by severe burn injury.

It has been well documented that many critical surgical diseases, such as shock, trauma and burn injury, causes the disruption of intestinal epithelial barrier function, leading to the leakage of bacteria, microbial products or other antigens from the intestinal lumen into the mucosa or systemic circulation to initiate or exacerbate an inflammatory response [1], [2], [4]. Here, we demonstrate that intestinal epithelial permeability to 4.4 kDa FITC-dextran starts to increase at 1 hour, peaks at 6 hours, and is still higher than sham burn at 24 hours following burn injury. This time-course pattern of postburn intestinal permeability is similar to a recently published study which reveals intestinal permeability to 4 kDa FITC-dextran peaks at 4 hours and returns to baseline at 24 hours following severe burn [19]. In addition, our present data show that the burn-caused increase of intestinal permeability is accompanied by the histological damage of intestinal mucosa, which is consistent with other previous studies [10], [19]. Taken together, an intact intestinal epithelial barrier function is disrupted after severe thermal injury.

Given that the intestinal barrier disruption is induced following severe burn, the underlying mechanisms are still incompletely elucidated. Actually, the early systemic and intestinal damage caused by thermal injury is orchestrated by a series of pathophysiological events such as ischemia, hypoxia, and inflammation, which have been demonstrated to contribute to intestinal barrier dysfunction. We and others have previously shown that hypoxia or ischemia, which occurs rapidly following severe burns, is capable of causing barrier disruption [20]–[23]. Furthermore, it has been well documented that some proinflammatory cytokines such as TNF-α, IL-1β, IL-6 and IFN-γ are significantly up-regulated in severely burned mice, rats and patients [24]–[26]. These up-regulated proinflammatory cytokines may contribute to the burn-induced intestinal barrier disruption. We, along with other investigators, have previously revealed that proinflammatory cytokines, including IFN-γ, TNF-α, IL-1β, IL-6, IL-13 and TNF superfamily member LIGHT, are able to induce intestinal epithelial barrier defects [14]–[18], [27]–[30]. Hence, it is reasonable to suggest that ischemia, hypoxia and inflammation contribute to the intestinal epithelial barrier breakdown induced by severe burn injury.

Intestinal barrier function disruption is characterized by the increased paracellular permeability as well as the changes of tight junction protein expression and organization [31], [32]. Previously published data have demonstrated that hypoxia and proinflammatory cytokines are able to induce the relocalization of tight junction proteins [14]–[16], [21], [33]. Here, we show that the intestinal barrier defects following severe burn injury is accompanied by the reorganization of tight junction proteins ZO-1, occludin and claudin-1. This is similar to the previous study revealing that both decreased expression and reorganization of intestinal tight junction proteins ZO-1 and occludin are induced in balb/c mice undergoing a 30% TBSA steam burn [34]. However, it has been reported that both mRNA and protein expression of occludin are up-regulated in Wistar rats inflicted with 30% TBSA scald injury [35]. Thus, it is suggested that the reorganization of tight junction proteins is involved in the burn-induced intestinal barrier disruption, whereas the role of altered expression of tight junction proteins is still controversial, and need to be defined.

MLCK activation, which directly leads to the phosphorylation of MLC, has been viewed as a common final pathway of acute tight junction regulation in response to a broad range of stimuli [7], [8]. It has been reported that MLCK activation alone is sufficient to increase tight junction permeability, which is associated with biological and morphological reorganization of the tight junction proteins ZO-1 and occludin [9]. Our previous *in vitro* studies have shown that increased MLC phosphorylation mediated by upregulated MLCK protein expression is critical to barrier breakdown induced by hypoxia or proinflammatory cytokines [14]–[16], [21], [22], [30], and that the barrier dysfunction is prevented by pharmacological MLCK inhibition [14], [21], [22], [30]. In this study, we demonstrate that after severe burn injury, intestinal barrier breakdown is accompanied by the upregulation of both MLCK protein expression and MLC phosphorylation in ileal mucosa. In addition, MLCK inhibition with ML-9, an MLCK inhibitor that is known to block MLCK activity [21], [22], [36], [37], not only abolishes the burn-induced increase of MLC phosphorylation in ileal mucosa, but also attenuates the increased intestinal permeability, histological damage of mucosa, as well as reorganization of tight junction protein ZO-1 following severe thermal injury. Thus, it is indicated that MLCK-dependent MLC phosphorylation signaling pathway is involved in the intestinal barrier disruption induced by severe burn injury.

It should be noted that MLCK inhibition with ML-9 just ameliorates, but not corrects, the burn-caused intestinal barrier breakdown. Thus, other signaling pathways should be considered in the pathogenesis of intestinal barrier disruption following severe burn injury. For example, the accumulating published data have shown that the percentage of apoptotic intestinal epithelial cells is significantly increased in mice suffering from 30% TBSA cutaneous full-thickness burn [38]–[40]. Our previous studies have also shown that the increase of apoptosis is accompanied by the increased Caspase-3 activity and the decreased Bcl-2 expression in intestinal epithelial cells after 30% TBSA burn injury [41]–[43]. Although the relevance of single-cell apoptosis to intestinal barrier dysfunction remains controversial owing to differing results in diverse experimental systems [44]–[48], extensive apoptosis of epithelial cells may lead to intestinal barrier dysruption. Therefore, the burn-induced increase in epithelial cell apoptosis may contribute to intestinal barrier dysfunction following thermal injury. In addition, we have previously demonstrated that Rho-associated kinase is involved in the intestinal barrier dysfunction in rats undergoing 30% TBSA thermal injury, and that pharmacological inhibition of Rho-associated kinase attenuates the burn-induced intestinal barrier dysfunction [49]. Moreover, it has been reported that Toll-like receptor 4 and p38 mitogen-activated protein kinase signaling pathways also play critical roles in intestinal epithelial barrier disruption following severe burn injury [50], [51].

In summary, our present data demonstrate the involvement of MLCK-dependent MLC phosphorylation signaling pathway in intestinal barrier dysfunction following severe burn injury. To the best of our knowledge, this is the first *in vivo* experimental study providing direct evidence to show the role of MLCK-dependent MLC phosphorylation in burn-induced intestinal epithelial barrier disruption. It is suggested that MLCK-dependent MLC phosphorylation may be a critical target for the therapeutic treatment of intestinal epithelial barrier disruption after severe burn injury.
